# Assessing the effects of National Health Insurance reimbursement policy revisions for anti-osteoporotic drugs in Korean women aged 50 or older

**DOI:** 10.1371/journal.pone.0244759

**Published:** 2020-12-31

**Authors:** Ja Seo Koo, Seong Hwan Moon, Hankil Lee, Sohee Park, Yun Mi Yu, Hye-Young Kang

**Affiliations:** 1 Department of Pharmaceutical Medicine and Regulatory Sciences, Colleges of Medicine and Pharmacy, Yonsei University, Incheon, South Korea; 2 Department of Medical Affairs, Sanofi-aventis Korea Co., Ltd., Seoul, South Korea; 3 Department of Orthopedic Surgery, College of Medicine, Yonsei University, Seoul, South Korea; 4 Institute of Health Services Research, Yonsei University, Seoul, South Korea; 5 Graduate School of Public Health, Yonsei University, Seoul, South Korea; 6 College of Pharmacy, Yonsei Institute of Pharmaceutical Sciences, Yonsei University, Incheon, South Korea; Medical College of Wisconsin, UNITED STATES

## Abstract

**Introduction:**

The Korean National Health Insurance revised its reimbursement criteria to expand coverage for anti-osteoporotic drug treatments in 2011 (expanding diagnostic criteria and the coverage period for anti-osteoporotic therapy) and 2015 (including osteoporotic fracture patients regardless of bone mineral density). We examined whether the two revisions contributed to an increase in the prescription rates of anti-osteoporotic drugs in Korea.

**Methods:**

We used the Health Insurance Review and Assessment Service-National Patient Sample data from 2010 through 2016. A segmented regression analysis of interrupted time series was performed to assess changes in the monthly prescription rates of anti-osteoporotic drugs among women aged 50 or older, defined as the proportion of elderly women prescribed with anti-osteoporotic drugs.

**Results:**

Both the levels (i.e., abrupt jump or drop) and the trends (i.e., slope) of the prescription rates of anti-osteoporotic drugs in the general population, osteoporotic patients, and osteoporotic fracture patients showed no significant changes after the first revision. However, there was a significant increase in the trends in the general population (β = 0.0166, p = 0.0173) and in osteoporotic patients (β = 0.1128, p = 0.0157) after the second revision. Women aged 65 to 79 years were the most significantly increased group in terms of the treatment proportion after the second revision because the trend was significant after the second revision in all three study populations (β = 0.0300, 0.1212, 0.1392, respectively; p < 0.05).

**Conclusions:**

Although the two revisions expanded reimbursement coverage, only the second revision on reimbursing based on osteoporotic fracture regardless of bone mineral density was associated with increasing the proportion of post-menopausal women being treated with anti-osteoporotic drugs.

## Introduction

Osteoporosis, characterized by a reduced density and quality of bone, is one of the major chronic conditions in the elderly population and is a significant risk factor for fractures among elderly individuals. The International Osteoporosis Foundation estimates that over 200 million women worldwide suffer from osteoporosis—approximately one-tenth of women aged 60, one-fifth of women aged 70, two-fifths of women aged 80, and two-thirds of women aged 90 years. Moreover, one of three women over 50 years of age will experience osteoporotic fractures. The incidences of osteoporosis and fracture increase with age and elderly osteoporotic women are at high risk of fracture [1]. It is important to treat osteoporosis to prevent fractures in postmenopausal women because it was reported that single bone mineral density (BMD) and past fracture were predictors of long-term fracture incidence [2]. While bisphosphonates as mainly used in anti-osteoporotic therapy show evidence for “broad-spectrum” anti-fracture efficacy, concern about atypical femur fractures occurrence after treatment was addressed. Therefore, anti-osteoporotic drug therapy should be used according to the benefit/harm ratio in individual patients [3,4].

With an increasing awareness that osteoporosis is a major risk factor for fractures in the elderly, the Korean National Health Insurance (NHI) after 1998 began to reimburse costs for anti-osteoporotic drug treatments to prevent osteoporotic fractures. However, the reimbursement criterion for anti-osteoporotic drugs, which was a T-score ≤ −3.0, was below the diagnostic criteria of osteoporosis (i.e., a T-score ≤ −2.5) [5]. Also, anti-osteoporotic drugs were reimbursed for only six months, although long-term drug therapy is necessary [6–9]. Such limited reimbursement by the NHI was criticized because prescription of anti-osteoporotic drugs applying such criteria was not effective in preventing osteoporotic fractures.

**Fig 1:**
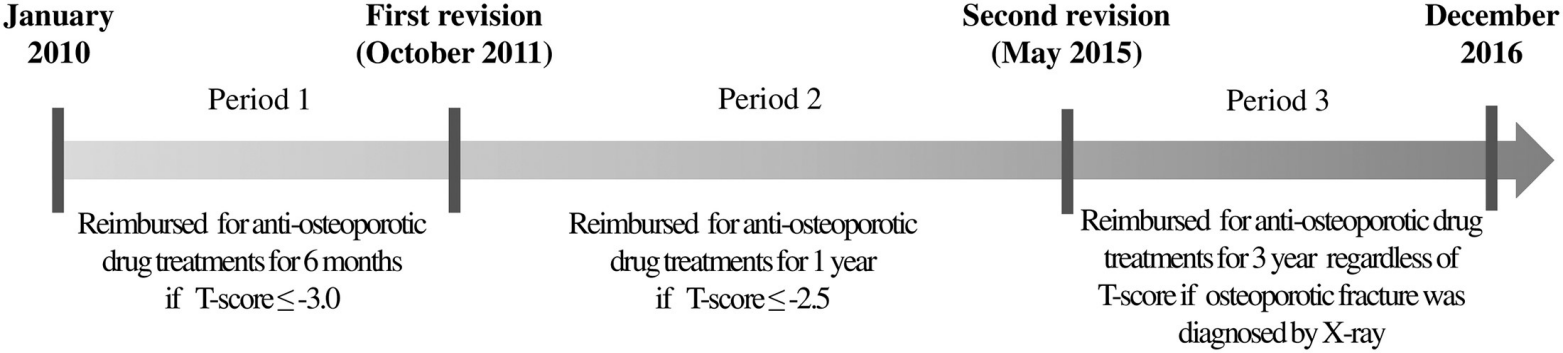
Revisions of the National Health Insurance reimbursement guideline for anti-osteoporotic drugs from 2010 to 2016.

In response to these concerns, the NHI reimbursement guidelines for anti-osteoporotic drugs were revised in October 2011 (first revision) and then in May 2015 (second revision), with the aim of preventing primary and secondary osteoporotic fractures in an aging society. In the first revision, the reimbursement criterion and period for anti-osteoporotic drug treatments were respectively expanded from T-score ≤ −3.0 to ≤ −2.5 and from six months to one year, in order to treat osteoporotic patients earlier and allow for more prolonged therapy. Through the second revision, the anti-osteoporotic drug could be reimbursed for three years, regardless of the level of the T-score, if an osteoporotic fracture was diagnosed by X-ray (Fig 1).

However, there has been no empirical study to assess the effectiveness of the revised NHI reimbursement guidelines for anti-osteoporotic drugs in Korea. Therefore, we conducted this study, analyzing the changes of prescription rates of anti-osteoporotic drugs after the revision of the reimbursement guidelines, to examine whether the NHI reimbursement expansion was associated with improving the opportunities for Korean women with osteoporosis to receive drug treatment.

## Materials and methods

### Data sources

This study used data from the Health Insurance Review and Assessment Service-National Patient Sample (HIRA-NPS) in Korea from 2010 through 2016. Each year, a 3% random sample (approximately 1.4 million persons) of the entire population, stratified by the total of 32 groups according to sex (2 strata) and age (16 strata), are selected, and their NHI claims records from the relevant year are included in the HIRA-NPS data. All patient data in HIRA-NPS were fully anonymized to ensure privacy [10]. Korea has a National Health Security System with two tiers: the NHI and Medical Aid (MA) programs (97% and 3% of the population, respectively). Both NHI and MA beneficiaries are sampled in HIRA-NPS, and the reimbursement revision for anti-osteoporotic drug therapy was applied both to the NHI and to the MA beneficiaries [11]. The research protocol was approved by the Institutional Review Board of Yonsei University (IRB No. 7001988-201803-HR-329-01E). The need for informed consent from the study population was waived by the board.

### Study population

Patients with osteoporosis and/or osteoporotic fracture are directly benefited by the changes of the first and second reimbursement policies. Therefore, we analyzed the impact of the policy among three different study populations. First, women aged 50 years or older as a reference were defined as the general population (GP). Second, women aged 50 years or older with a record of a diagnosis of osteoporosis [International Classification of Diseases code, 10th revision (ICD-10): M80, M81, and M82] were defined as osteoporotic patients (OP). Third, women aged 50 years or older with a record of a diagnosis of osteoporotic fracture were defined as osteoporotic fracture patients (OFP). Based on previous studies that defined osteoporotic fractures using diagnosis codes from claims records [12–16], we defined the following fractures as osteoporotic fractures: hip fractures (S72.0 and S72.1); spine fractures (S22.0, S22.1, S32.0, M48.4 and M48.5); distal radius fractures (S52.5 and S52.6); cervical fractures (S42.0); humerus fractures (S42.2 and S42.3); and distal tibia fractures (S82.3, S82.5 and S82.6).

Anti-osteoporotic drugs in this study included drugs indicated for osteoporosis treatment under the approved label and reimbursed by NHI during the study period. Specific drugs included the following: bisphosphonates (alendronate, etidronate, ibandronate, pamidronate, risedronate and zoledronate); bisphosphonate/vitamin D combination (alendronate/calcitriol, alendronate/cholecalciferol, ibandronate/cholecalciferol and risedronate/cholecalciferol); selective estrogen receptor modulator (bazedoxifene and raloxifene); and active vitamin D (alfacalcidol and calcitriol). We searched approved drugs indicated for osteoporosis treatment in the drug database of the Ministry of Food and Drug Safety and in the reimbursement guidelines for anti-osteoporotic drugs. Using nine-digit Korean Active Pharmaceutical Ingredients codes listed by the HIRA, we identified specific anti-osteoporotic drugs from the HIRA-NPS data for the analysis.

### Measurements and data analysis

Although the ultimate goal of the policy was to prevent osteoporotic fractures, it is difficult to measure the effectiveness of the policy in terms of the reduction of the incidence of osteoporotic fracture due to the long lag time between the drug therapy and its outcome of osteoporotic fracture prevention. Thus, alternatively, we chose to examine the prescription rate of anti-osteoporotic drugs, which is a process measure that aims to capture the impact of the policy within a short-term observation period following the policy change. We analyzed whether the prescription rate, measured as the proportion of elderly women treated with anti-osteoporotic drugs in each study group, increased after the revision. Time series data were constructed by calculating the prescription rates at monthly time points during the study period (S1 Table).

If individuals had claim records with prescriptions for anti-osteoporotic drugs, we considered them as being treated with anti-osteoporotic drugs. As a first step to investigate whether there were noticeable changes in the prescription rates of anti-osteoporotic drugs following the first and second reimbursement guideline revisions, in October 2011 and May 2015, we visually inspected a plot of the prescription rate over time. More specifically, we inspected a change in the levels of prescription rate (i.e., an abrupt jump or drop) after the implementation of each revision. Also, we looked for a change in the trend of the prescription rates by looking for increases or decreases in the slope of the segment after the revision as compared with the segment preceding the revision.

To test whether the identified changes in the level and the trend of the prescription rates were the result of chance or not, we conducted a segmented regression analysis of an interrupted time series. Segmented regression analysis of interrupted time series is a powerful statistical method to assess “how much an intervention changed an outcome of interest, immediately and over time; instantly or with delay; and transiently or long-term.” This statistical approach is appropriate if data can produce summarized outcome measures at regular, evenly spaced intervals. However, its limitations include that changes may follow non-linear patterns despite assuming a linear trend and individual-level covariates are not controlled [17]. Because the effect of intervention and persistence over time can be confirmed by comparing levels and trends measured in intervals, segmented regression analysis of interrupted time series has been employed frequently in assessing policy effects over time [18–20]. Since our study had access to time series data with monthly prescription rates of anti-osteoporotic drugs for 84 consecutive months from January 2010 through December 2016, we were able to assess the impact of the reimbursement expansions using this approach.

We assessed the impacts of the first and the second revisions in the GP, OP, and OFP groups. Because there are seasonal variations in the incidence of osteoporotic fracture [21–23], we assumed that the prescription rates of anti-osteoporotic drugs would have seasonal variations as well. To control for the effect of seasonality on the dependent variable, we included dummy variables for the seasons in the regression models. Furthermore, the autocorrelation of error terms was detected by the Durbin-Watson test [17], and thus an autoregressive integrated moving average (ARIMA) model with a second-order difference of independent variables was used to adjust the first-order autocorrelation. The statistical package SAS System for Windows, version 9.4 (SAS Inc., Cary, NC, USA) was used to perform the analyses in this study.

Impacts of reimbursement guideline revisions may vary across different age groups because osteoporosis and osteoporotic fractures are more prevalent in older groups. Thus, we stratified the segmented regression analysis and compared the impacts of the reimbursement guideline revisions among three age groups: 50–64, 65–79, and ≥ 80 years old.

## Results

Among the 1,819,062 women aged 50 years or above included in the 2010–2016 HIRA-NPS data, 408,759 OP (22.5%) and 79,451 OFP (4.4%) were identified. The number of patients prescribed anti-osteoporotic drugs was 211,384 (51.7%) in OP and 17,523 (22.1%) in OFP.

Table 1 shows the baseline characteristics of the study population in January 2010. The highest proportions among the three age groups were observed in the 65–79 years age group of OP (57.56%) and OFP (50.02%), and the 50–64 years age group of GP (53.29%). The majority of the patients were enrolled in the NHI (92.59% among GP, 86.75% among OP and 88.37% among OFP). The proportions in medical institutions and specialty areas were slightly different across the three study population groups due to disease characteristics. The proportion of patients who were treated in the clinic was higher in GP (77.52%) and in OP (73.78%) than in OFP (58.16%). Also OFP were mainly treated by an orthopedics department (48.59%), while GP and most of the OP were treated by internal medicine (38.41% and 33.40%, respectively).

**Table 1:** Baseline characteristics of patients with osteoporosis and osteoporotic fracture (January 2010).

	General population		Osteoporotic patients		Osteoporotic fracture patients	
	N	%	N	%	N	%
Total	161,306	100.00	16,387	100.00	2,519	100.00
Age (years)						
50–64	85,959	53.29	5,230	31.92	735	29.18
65–79	61,842	38.34	9,432	57.56	1,260	50.02
≥80	13,505	8.37	1,725	10.53	524	20.80
Type of National Health Security Program enrolled						
National Health Insurance	149,357	92.59	14,215	86.75	2,226	88.37
Medical aid	11,949	7.41	2,172	13.25	293	11.63
Type of health care institution treating osteoporosis or osteoporotic fracture						
Tertiary hospital	7,176	4.45	867	5.29	126	5.00
General hospital	10,428	6.46	1,468	8.96	388	15.40
Hospital	10,486	6.50	1,403	8.56	510	20.25
Clinic	125,040	77.52	12,090	73.78	1,465	58.16
Health center	8,176	5.07	559	3.41	30	1.19
Specialty of clinicians treating osteoporosis or osteoporotic fracture						
Internal medicine	61,954	38.41	5,473	33.40	449	17.82
Orthopedics	23,515	14.58	4,441	27.10	1,224	48.59
Family medicine	5,371	3.33	655	4.00	69	2.74
Neurosurgery	2,810	1.74	538	3.28	196	7.78
Others	67,656	41.94	5,280	32.22	581	23.07

**Fig 2:**
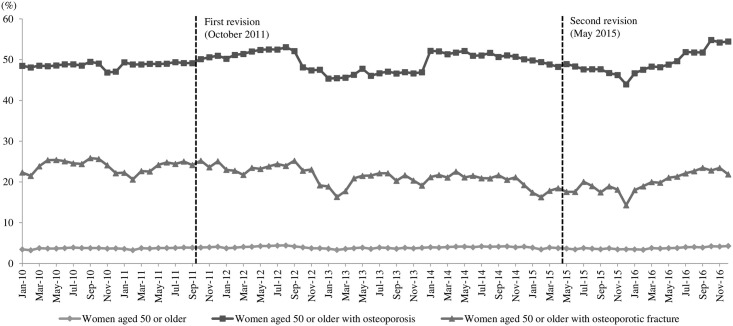
Prescription rates of anti-osteoporotic drugs in women aged 50 or older in Korea.

Fig 2 presents the monthly prescription rates from 2010 through 2016, with 84 data points that were defined as the proportion of women prescribed anti-osteoporotic drugs. Through direct observation, we can see that the proportion of OP treated with anti-osteoporotic drugs increased for approximately one year after the first revision and then decreased to the level seen before the first revision. About 6 months after the second revision, the prescription rate began to increase, and the increasing trend continued for one year until the censoring point of December 2016. A different trend was observed for OFP. There was no increase in the proportion of OFP treated by anti-osteoporotic drugs after the first revision, and, instead, the proportion continued to decrease. However, similar to the OP, the prescription rate started increasing about 6 months after the second revision and continued to increase.

Table 2 presents the regression analysis results of our study. For all three populations, there was no significant month-to-month change in the prescription rate of anti-osteoporotic drugs before the first revision [p-value for baseline trend (*β*_*1*_): 0.6215 for GP, 0.6046 for OP and 0.4416 for OFP, respectively]. There was no significant change in the prescription rate of anti-osteoporotic drugs after either the first [p-value for level change (*β*_*2*_): 0.3891, 0.9067 and 0.7070, respectively] or the second revision [p-value for level change (*β*_*4*_): 0.2454, 0.2734 and 0.6063, respectively]. While there was no significant change in the month-to-month trend in the prescription rate of anti-osteoporotic drugs after the first revision [p-value for trend change (*β*_*3*_): 0.3999, 0.5081 and 0.7297, respectively], there was a significant increase after the second revision in trends for both GP and OP, but not for OFP [*β*_*5*_ = 0.0166, 0.1128 and 0.0931, respectively; and p-value for trend change (*β*_*5*_): 0.0173, 0.0157 and 0.0684, respectively].

**Table 2:** Results of the segmented regression analysis for the reimbursement revision’s effect on prescription rates of anti-osteoporotic drugs in women aged 50 or older.

	General population		Osteoporotic patients		Osteoporotic fracture patients	
	Coefficient	p-value	Coefficient	p-value	Coefficient	p-value
β_0_ (intercept)	0.0823	0.3092	-0.1183	0.8264	1.4143	0.0196
β_1_ (TIME1, baseline trend)	0.0030	0.6215	0.0211	0.6046	-0.0346	0.4416
β_2_ (POLICY1, level change after the 1^st^ revision)	0.0761	0.3891	0.0691	0.9067	0.2444	0.7070
β_3_ (TIME2, trend change after the 1^st^ revision)	-0.0054	0.3999	-0.0285	0.5081	0.0164	0.7297
β_4_ (POLICY2, level change after the 2^nd^ revision)	-0.1084	0.2454	-0.6834	0.2734	-0.3534	0.6063
β_5_ (TIME3, trend change after the 2^nd^ revision)	0.0166	0.0173	0.1128	0.0157	0.0931	0.0684
β_6_ (Season1)	-0.0524	0.3155	-0.1446	0.6779	-0.3535	0.3581
β_7_ (Season2)	-0.2301	< 0.0001	-0.5848	0.0968	-0.7803	0.0454
β_8_ (Season3)	-0.2243	< 0.0001	-0.0912	0.7922	-1.6506	< 0.0001
Durbin-Watson statistics	2.3769^a^		2.2387^a^		2.4372^a^	

^a^p-value for the hypothesis of positive autocorrelation: > 0.05, p-value for the hypothesis of negative autocorrelation: > 0.05.

Segmented regression analysis was performed for the three age subgroups (50–64, 65–79, and ≥ 80 years old), respectively, to identify which groups were impacted by the revision of the reimbursement guidelines (Table 3). None of the three age groups showed significant level or trend changes after the first revision. This implies that the first reimbursement guideline revision was not associated with expanding the number of people receiving anti-osteoporotic drug therapy within any age group. On the other hand, the second revision showed significant trend changes, with positive regression coefficients. In particular, in the 65–79 years old group, all three study populations had significant increases in their prescription rates during the period after the second revision (β_5_ = 0.0300, 0.1212, and 0.1392, respectively). The magnitude of the regression coefficient was greatest in OFP (β_5_ = 0.1392), followed by OP (β_5_ = 0.1212) and GP (β_5_ = 0.0300). For those above 80 years old, only OP had a significant increase in the prescription rate during the period after the second revision (β_5_ = 0.1496).

**Table 3:** Results of segmented regression analysis of reimbursement revision effects on prescription rates for anti-osteoporotic drugs, according to age group.

	50–64 years old			65–79 years old			≥80 years old		
	General population	Osteoporotic patients	Osteoporotic fracture patients	General population	Osteoporotic patients	Osteoporotic fracture patients	General population	Osteoporotic patients	Osteoporotic fracture patients
β_0_ (intercept)	0.0433	-0.1423	1.2561	0.1589	0.0718	1.5476	0.1060	0.1037	1.1959
β_1_ (TIME1, baseline trend)	0.0014	0.0205	-0.0397	0.0052	0.0097	-0.0350	0.0054	0.0229	-0.0254
β_2_ (POLICY1, level change after the 1^st^ revision)	0.0267	-0.0941	0.1354	0.1134	0.1782	0.1328	0.0003	-0.2215	0.3482
β_3_ (TIME2, trend change after the 1^st^ revision)	-0.0031	-0.0272	0.0272	-0.0090	-0.0219	0.0143	-0.0058	-0.0302	-0.0065
β_4_ (POLICY2, level change after the 2^nd^ revision)	-0.0384	-0.3819	-0.1769	-0.2239	-0.7701	-0.7622	-0.1394	-0.7558	-0.1181
β_5_ (TIME3, trend change after the 2^nd^ revision)	0.0099^a^	0.0896	0.0880	0.0300^a^	0.1212^a^	0.1392^a^	0.0222	0.1496^a^	0.1035
β_6_ (Season1)	0.0033	-0.1904	-0.1411	-0.1204	-0.0309	-0.3141	-0.0590	-0.0324	-0.4197
β_7_ (Season2)	-0.1006^a^	-0.1086	-0.3674	-0.4011^a^	-0.6998	-0.8379	-0.3690^a^	-1.1044^a^	-0.6685
β_8_ (Season3)	-0.0931^a^	0.2886	-1.3283^a^	-0.3942^a^	-0.1146	-1.8482^a^	-0.2673^a^	-0.3593	-0.8996
Durbin-Watson statistics	2.3761^b^	2.1839^b^	2.3378^b^	2.3157^b^	2.2059^b^	2.3851^b^	2.3594^b^	2.2940^b^	2.3011^b^

^a^p < 0.05.

^b^p-value for the hypothesis of positive autocorrelation: > 0.05, p-value for the hypothesis of negative autocorrelation: > 0.05.

## Discussion

In this study, we evaluated the impacts of NHI reimbursement guideline revisions for anti-osteoporotic drug therapy in Korea, which were intended to expand the NHI coverage for osteoporotic patients with the goal of preventing osteoporotic fractures throughout the nation. The segmented regression analysis results showed that the first revision, which expanded the diagnostic criteria of osteoporosis from a T-score of −3.0 to −2.5 and increased the reimbursement period from 6 months to 1 year after diagnosis, was not associated with expanding the number of post-menopausal women aged 50 or older receiving anti-osteoporotic drug therapy. This result implies that the first revision did not lead to an increase in the population benefitting from osteoporosis treatment, and therefore it may not be an effective policy for reducing the number of osteoporotic fractures in society.

The underlying reasons for this finding could be explained by the following. First, the Korean NHI reimburses BMD tests performed to diagnose osteoporosis only for women aged 65 or older and men aged 70 or older [12]. Because women between the ages of 50 and 64 are not subject to the reimbursement for the BMD test, they have a low chance to be diagnosed and to have an opportunity to get treated. Second, the first revision set a restriction that BMD had to be measured by DXA or quantitative computed tomography (QCT) tests on the central bone. However, most osteoporotic patients in Korea are diagnosed by quantitative ultrasonogram (QUS) in clinics. Thus, even if the government relaxed the reimbursement criteria of osteoporosis from a T-score of −3.0 to −2.5, the reimbursement restriction on the diagnostic tests is an obstacle to receiving anti-osteoporotic drug therapy.

Unlike the first revised guideline, the second revision, which reimburses anti-osteoporotic drug treatment for three years if the osteoporotic fracture was diagnosed by X-ray regardless of the level of the T-score, appeared to be successful in increasing the number of patients treated with anti-osteoporotic drug therapy. In women aged 50 or older, significant trend changes in the prescription rates for anti-osteoporotic drugs were observed for GP (ß_5_ = 0.0166; p = 0.0173) and for OP (ß_5_ = 0.1128; p = 0.0157) after the implementation of the second guideline revision. Our analysis suggests that the second revision has had the strongest impact on increase of anti-osteoporotic drugs in women aged between 65 and 79 years. All three study populations showed significant increases after the revision in the populations treated with anti-osteoporotic drugs (ß_5_ = 0.0300, 0.1212 and 0.1392, and p = 0.0109, 0.0124 and 0.0483, respectively). This finding implies that a powerful driver in reimbursement guidelines to prescribe anti-osteoporotic drugs seems to be a fracture rather than t-score.

We performed a sensitivity analysis stratified according to the four types of anti-osteoporotic drugs (i.e. bisphosphonates; bisphosphonate/vitamin D combination; selective estrogen receptor modulator; and active vitamin D), in order to determine which specific anti-osteoporotic drugs were most impacted by the revisions, and to assess the robustness of the primary findings. Separate segmented regression analyses were performed with prescription rates of each of the four drug classes set as dependent variables. As a result, only the prescription rate of bisphosphonates among the four drug classes showed a significant trend change after the second revision in general population (β_5_ = 0.0119, p = 0.0138) and in osteoporotic patients (β_5_ = 0.0695, p = 0.0470). This result is similar to the primary findings, confirming the robustness of the primary findings. Because bisphosphonates were the most frequently prescribed anti-osteoporotic drugs in all of the three study populations during the study period (i.e., 56.0%-60.4% of prescribed anti-osteoporotic drugs), it seems that the use of bisphosphonates was a main driver in the prescription of anti-osteoporotic drugs among Korean elderly women.

Changes or external factors may potentially affect the prescription rates of anti-osteoporotic drugs other than the reimbursement revisions, per se during the study period, which might impact the robustness of the primary findings of this study. First, several anti-osteoporotic drugs are indicated for reasons other than osteoporosis. This might affect prescription rates of anti-osteoporotic medications during the study period and distort the study results. To examine this issue, we analyzed the proportions of prescriptions of anti-osteoporotic drugs with indications for other conditions than osteoporosis, which include some bisphosphonates such as etidronate, pamidronate and zoledronate. The proportion of prescriptions for those drugs relative to all drugs was 2.8% in the general population, 2.8% in the osteoporotic patients, and 4.3% in osteoporotic fracture patients, respectively. Because their proportion was very low, we believed that the impact of these products on our study results would be very marginal. Second, we checked the impact of new anti-osteoporotic drugs launched in the study period: bazedoxifene in 2012 and ibandronate/cholecalciferol in 2013. The proportion of prescriptions of those drugs out of all anti-osteoporotic drugs was very low (less than 5% even after the second revision). Thus, we considered that the primary results of our study might not be affected by the introduction of these new drugs. Third, BMD should be measured by DXA or QCT in order to get reimbursed for anti-osteoporotic drugs. The prescription rates of anti-osteoporotic drugs can be increased if the frequency of BMD tests increases. According to the analysis, the rates of overall BMD testing in women aged 50 or older were similar after the first (96.8%) and second revision (95.4%) respectively. Since the rate of BMD examination tests were maintained during the study period, we believe that the potential confounding effect of the increase in the rate of BMD tests on the prescription rate of anti-osteoporotic drugs during the study period would be very low.

There are a number of studies that have evaluated the effects of changes in reimbursement guidelines for diagnostic tests or treatment for osteoporosis [24–26]. For example, in 2007, Australia’s Medicare initiated reimbursement for DXA tests for elderly patients aged 70 or older, designated as a high-risk group for fractures, in order to delay or prevent the occurrence of fractures. The effect of this policy was evaluated by examining the changes in the proportion of DXA tests and the incidence of fractures in two separate studies. When comparing DXA test use before (2003–2006) and after (2007–2010) the introduction of the DXA reimbursement guideline, the proportion of DXA referrals in both men and women significantly increased, but that increase was small [24]. The changes of reimbursement guidelines had little impact in reducing fracture incidence in elderly men and women, as fracture rates increased in 2012 as compared to 2006 [25]. The Province of Ontario in Canada also changed its reimbursement policy for DXA tests in 2008. For the low-risk group, their NHI covered a DXA test every two to three years, while it was covered annually for the high-risk group. Jaglal et al. compared DXA test rates in adults aged 40 and older before and after the policy revision. While the reduction in testing for low-risk groups was mainly a result of the effects intended by the policy, it also showed a negative effect on the decrease among women within high-risk populations [26].

In those studies, the effects of changes of reimbursement guidelines for DXA tests to diagnose osteoporosis were primarily assessed from the rates of DXA testing, which is a process measure similar to the prescription rate measured in our study. Although the outcome measure, such as the rate of prevention of osteoporotic fracture, could be a more valid measure, it requires long-term follow-up observation. Thus, process measures, often defined as health care utilization rates rather than health outcomes, are used as an alternative method.

This study has several limitations. First, since this study used HIRA-NPS, the study subjects were included based on the diagnosis records in the claims data. There is a possibility that healthcare professionals entered diagnostic codes apart from the presence of the actual disease to obtain reimbursement, which is an intrinsic limitation of administrative claims data and may produce a bias. According to previous studies which assessed accuracy, correspondence between the major diagnosis of Health Insurance claims and medical records in Korea were 69.7% and 60.0% respectively [27,28]. Therefore, we may not rule out the possibility of discrepancy in this study. Second, it included data for 20 months after the second guideline revision because only data through 2016 were available publically. It may be difficult to determine whether that is enough time to confirm the effects of the reimbursement guideline revision in May 2015. However, if we extend the observation period further, it will be hard to distinguish between the effect of the reimbursement guideline revision and that of the introduction of new anti-osteoporotic drugs to the market on the increase in the prescription rates. For example, the NHI started providing reimbursement for new anti-osteoporotic drugs, such as teriparatide and denosumab, from December 2016 and October 2017, respectively. Third, the impact of the policy in this study was assessed with the number of patients treated with anti-osteoporotic drugs as a process measure because the period following the revision of the second reimbursement guidelines was too short for assessing actual measurements of fractures as outcomes. Fourth, an increasing rate of prescription of anti-osteoporotic drugs was found after the second revision in May 2015, but not after the first revision in 2011. There could be alternative explanation for this finding. For example, it is possible that both healthcare providers and patients became increasingly aware of the need for anti-osteoporosis treatment during the 5-year period, and this increased awareness led to more adherence to the reimbursement guidelines after the second revision. This might potentially confound the study findings. Lastly, we attempted to confirm the impact of the reimbursement revisions with multiple comparisons by analyzing three study populations separately, by conducting stratified analysis according to the three age groups, and by analyzing each of the four drug classes separately. However, there is a possibility that our analysis did not account for multiple comparisons that might affect the robustness of the primary findings. For example, the impact of the revisions may vary across different types of healthcare organizations (i.e., clinics, general hospitals, or tertiary care hospitals), different regions (i.e., big cities or small towns), or different types of programs enrolled under the National Health Security (i.e., National Health Insurance or Medical Aid). In future studies, more subgroup analyses or multiple comparisons may help confirm the robustness of our findings and in identifying target groups that do not benefit from the revision and are therefore in the need of other interventions to help them access anti-osteoporotic drug treatments.

In summary, this study demonstrated the first reimbursement guideline revision that expanded diagnostic criteria for osteoporosis and extended the coverage period for anti-osteoporotic therapy was not associated with expanding the proportion of post-menopausal women treated with anti-osteoporotic drugs. However, the second revision, which enlarged the eligibility pool for reimbursement and further extended the coverage period for anti-osteoporotic drug therapy, was associated with increase of anti-osteoporotic drugs. Although the second revision allowed osteoporotic fracture patients to receive coverage for drug treatment for three years, regardless of T-score, its association with increasing the proportion of osteoporotic fracture patients treated with anti-osteoporotic drugs was observed only for women aged between 65 and 79 years. Thus, the impact of the policy was demonstrated in a limited population. Long-term evaluation of the policy’s effect may be helpful to attain a more conclusive assessment of the revision.

## Supporting information

S1 Table Data structure for segmented regression analysis to analyze the impact of two policy changes on the monthly prescription rate of anti-osteoporotic drugs. (PDF)
