# Supplementary material for: Assessing the effects of National Health Insurance reimbursement policy revisions for anti-osteoporotic drugs in Korean women aged 50 or older
Source: PLoS One. 2020 Dec 31;15(12):e0244759. doi: 10.1371/journal.pone.0244759 (PMC7774923; doi:10.1371/journal.pone.0244759)
Supplement: S1 Table — (PDF) [file pone.0244759.s001.pdf]

S1 Table. Data structure for segmented regression analysis to analyze the impact of two policy changes on the monthly prescription rate of anti-osteoporotic drugs

Observation	Prescription rate of anti-osteoporotic drug %			Time1	Policy1	Time2	Policy2	Time3	Season1	Season2	Season3
	General population	Osteoporotic patients	Osteoporotic fracture patients								
1	4.93	48.49	22.31	1	0	0	0	0	0	0	1
2	4.73	48.10	21.49	2	0	0	0	0	0	0	1
3	5.19	48.54	23.85	3	0	0	0	0	0	0	0
4	5.07	48.40	25.34	4	0	0	0	0	0	0	0
5	5.09	48.59	25.39	5	0	0	0	0	0	0	0
6	5.24	48.86	25.09	6	0	0	0	0	1	0	0
7	5.42	48.87	24.56	7	0	0	0	0	1	0	0
8	5.27	48.56	24.41	8	0	0	0	0	1	0	0
9	5.20	49.48	25.85	9	0	0	0	0	0	1	0
10	5.14	49.05	25.65	10	0	0	0	0	0	1	0
11	4.98	46.85	24.08	11	0	0	0	0	0	1	0
12	4.90	47.06	22.14	12	0	0	0	0	0	0	1
13	5.01	49.36	22.26	13	0	0	0	0	0	0	1
14	4.79	48.83	20.61	14	0	0	0	0	0	0	1
15	5.13	48.83	22.66	15	0	0	0	0	0	0	0
16	5.08	49.00	22.57	16	0	0	0	0	0	0	0

17	5.22	48.93	24.19	17	0	0	0	0	0	0	0
18	5.26	49.03	24.79	18	0	0	0	0	1	0	0
19	5.35	49.40	24.41	19	0	0	0	0	1	0	0
20	5.41	49.18	25.01	20	0	0	0	0	1	0	0
21	5.29	49.16	24.14	21	0	0	0	0	0	1	0
22	5.38	50.14	25.21	22	1	1	0	0	0	1	0
23	5.41	50.59	23.58	23	1	2	0	0	0	1	0
24	5.48	50.97	25.06	24	1	3	0	0	0	0	1
25	5.29	50.24	22.96	25	1	4	0	0	0	0	1
26	5.49	51.17	22.75	26	1	5	0	0	0	0	1
27	5.67	51.41	21.72	27	1	6	0	0	0	0	0
28	5.78	52.02	23.47	28	1	7	0	0	0	0	0
29	5.94	52.40	23.20	29	1	8	0	0	0	0	0
30	6.10	52.53	23.82	30	1	9	0	0	1	0	0
31	6.26	52.48	24.38	31	1	10	0	0	1	0	0
32	6.32	53.06	23.94	32	1	11	0	0	1	0	0
33	5.99	52.12	25.21	33	1	12	0	0	0	1	0
34	5.50	48.13	22.77	34	1	13	0	0	0	1	0
35	5.27	47.39	23.05	35	1	14	0	0	0	1	0
36	5.25	47.54	19.17	36	1	15	0	0	0	0	1

37	5.08	45.37	18.89	37	1	16	0	0	0	0	1
38	4.82	45.45	16.31	38	1	17	0	0	0	0	1
39	5.04	45.60	17.73	39	1	18	0	0	0	0	0
40	5.23	46.30	20.89	40	1	19	0	0	0	0	0
41	5.45	47.78	21.50	41	1	20	0	0	0	0	0
42	5.23	46.05	21.57	42	1	21	0	0	1	0	0
43	5.51	46.67	22.13	43	1	22	0	0	1	0	0
44	5.40	47.08	22.14	44	1	23	0	0	1	0	0
45	5.17	46.62	20.28	45	1	24	0	0	0	1	0
46	5.37	46.96	21.64	46	1	25	0	0	0	1	0
47	5.25	46.64	20.36	47	1	26	0	0	0	1	0
48	5.35	46.93	19.11	48	1	27	0	0	0	0	1
49	5.68	52.16	21.19	49	1	28	0	0	0	0	1
50	5.58	52.05	21.69	50	1	29	0	0	0	0	1
51	5.72	51.36	21.08	51	1	30	0	0	0	0	0
52	5.88	51.75	22.54	52	1	31	0	0	0	0	0
53	5.93	52.15	21.11	53	1	32	0	0	0	0	0
54	5.84	50.97	21.51	54	1	33	0	0	1	0	0
55	5.98	51.04	20.90	55	1	34	0	0	1	0	0
56	5.94	51.68	20.88	56	1	35	0	0	1	0	0

57	5.88	50.66	21.64	57	1	36	0	0	0	1	0
58	5.89	51.06	20.53	58	1	37	0	0	0	1	0
59	5.65	50.71	21.14	59	1	38	0	0	0	1	0
60	5.64	50.11	19.23	60	1	39	0	0	0	0	1
61	5.39	49.82	17.37	61	1	40	0	0	0	0	1
62	4.97	49.43	16.25	62	1	41	0	0	0	0	1
63	5.31	48.83	17.84	63	1	42	0	0	0	0	0
64	5.19	48.26	18.47	64	1	43	0	0	0	0	0
65	5.14	48.94	17.58	65	1	44	1	1	0	0	0
66	5.04	48.355	17.58	66	1	45	1	2	1	0	0
67	5.36	47.62	20.05	67	1	46	1	3	1	0	0
68	5.15	47.66	19.00	68	1	47	1	4	1	0	0
69	4.93	47.65	17.42	69	1	48	1	5	0	1	0
70	5.06	46.72	18.93	70	1	49	1	6	0	1	0
71	4.82	46.25	18.11	71	1	50	1	7	0	1	0
72	4.67	43.94	14.27	72	1	51	1	8	0	0	1
73	4.88	46.69	17.98	73	1	52	1	9	0	0	1
74	4.72	47.53	18.93	74	1	53	1	10	0	0	1
75	5.18	48.32	19.96	75	1	54	1	11	0	0	0
76	5.08	48.17	19.81	76	1	55	1	12	0	0	0

77	5.20	48.82	21.04	77	1	56	1	13	0	0	0
78	5.32	49.65	21.32	78	1	57	1	14	1	0	0
79	5.65	51.87	22.12	79	1	58	1	15	1	0	0
80	5.62	51.79	22.64	80	1	59	1	16	1	0	0
81	5.48	51.78	23.47	81	1	60	1	17	0	1	0
82	5.81	54.83	22.81	82	1	61	1	18	0	1	0
83	5.74	54.23	23.44	83	1	62	1	19	0	1	0
84	5.66	54.47	21.85	84	1	63	1	20	0	0	1
